# Accumulation Characteristics and Pollution Evaluation of Soil Heavy Metals in Different Land Use Types: Study on the Whole Region of Tianjin

**DOI:** 10.3390/ijerph191610013

**Published:** 2022-08-14

**Authors:** Tiantian Ma, Youwen Zhang, Qingbai Hu, Minghai Han, Xiaohua Li, Youjun Zhang, Zhiguang Li, Rongguang Shi

**Affiliations:** 1 Agro-Environmental Protection Institute, Ministry of Agriculture and Rural Affairs, Tianjin 300170, China; 2 School of Environmental Science & Safety Engineering, Tianjin University of Technology, Tianjin 300384, China; 3 Qamdo Institute of Agricultural Science, Qamdo 854000, China; 4 Rual Energy and Environment Agency, Ministry of Agriculture and Rural Affairs, Beijing 100125, China; 5 Tianjin North China Geological Exploration Bureau, 67, Guang-rui-xi-lu Rd., Tianjin 300170, China; 6 School of Environmental Science and Engineering, Tianjin University, 92, Weijin Rd., Nankai District, Tianjin 300350, China; 7 Agricultural Technology Extension Service Centre of Jingyuan County, Baiyin 730900, China

**Keywords:** soil, heavy metals, spatial distribution, source analysis, pollution assessment

## Abstract

Heavy metal pollution in soil has received much attention in recent decades. Many studies have analyzed the interaction between specific soil quality and soil heavy metal pollution. However, there is little information about the pollution status, spatial distribution and pollution sources of heavy metals in the province of Tianjin. In this paper, the distribution characteristics and pollution sources of heavy metals in soil were studied by means of the surface soil of Tianjin, as the study area and object, conducted in combination with land use types, using multiple data analysis and multivariate statistics, while the pollution levels were evaluated by various indices. The results showed the mean contents of the seven heavy metals of the studied elements followed an increasing order of Cd (0.15 mg/kg) < As (11.9 mg/kg) < Cu (24.3 mg/kg) = Pb (24.3 mg/kg) < Ni (27.9 mg/kg) < Cr (70.7 mg/kg) < Zn (79.1 mg/kg). The median values of Cr and Ni were lower than the background values and did not exceed the screening values at the points, and the median values of Cu, Zn and Pb were close to the background values, while the median contents of As and Cd were higher than the background values. The highest accumulation of heavy metals was found in grassland, and the coefficient of variation of heavy metal contents were higher in garden land, industrial and mining storage land, residential land and transportation land, indicating that the soil heavy metal contents under these land use types were more significantly disturbed by human factors. The evaluation results of the ground accumulation index method showed that the soil in Tianjin was free of pollution, except for Cd, which was at the non-polluted to moderately polluted level. The Nemero integrated pollution index evaluation method and the pollution load index evaluation method together showed that the integrated pollution levels of heavy metals in Tianjin soils were both at no pollution level/safety level. Apart from Cd and As, which were not correlated, the other heavy metals were correlated with each other two by two. Cd, Pb and Zn were the main pollution contributors from traffic, industry and other anthropogenic factors, while Cr and Ni were the main pollution contributors from soil parent material, and Cu was the main pollution contributor from mining and metal smelting. In addition, As was presumed to be the main source of pollution contribution from agriculture and surface runoff.

## 1. Introduction

With the rapid development of industry and urban construction and the improvement of peoples’ living standards, a large number of pollutants have been poured into the soil. A large quantity of heavy metals accumulates in the soil, causing serious impacts on the soil environment, and endangering the ecological environment and human health [1]. Previous studies have revealed that human exposure to metals, such As, Pb and Hg, lead to their accumulation in fatty tissues and affect the central nervous system [2,3]. A large amount of untreated sewage is discharged indiscriminately, industrial solid waste is piled up at will, and sewage irrigation, irrational application of agrochemicals, such as fertilizers, pesticides, plant growth regulators, and agricultural films, are associated with urban environmental quality, and, as a consequence, soil becomes heavily polluted and its ecosystem function extremely degraded [4]. It has been reported that, as of 2014, more than 70% of the soil (hundreds of thousands of sampling points) in the United States had been damaged by pollutants, while tens of thousands of hectares of arable land in China were also polluted by heavy metals [5]. Urban soils have some specific characteristics, such as unpredictable layering, poor structure, and high concentrations of trace elements [6]. Due to the high population density and intensive anthropogenic activities, urban soils have been severely disturbed. Consequently, a great number of environmental problems have emerged, among which heavy metal pollution remains a major issue. Therefore, research on the accumulation characteristics of heavy metals in urban soils and their pollution levels is of great significance for the treatment and prevention of heavy metal pollution and the improvement of peoples’ living quality [7].

Excessive heavy metal content in the soil causes soil dysfunction, deterioration of environmental quality, and reduces crop yields. Enrichment of heavy metals through the food chain eventually affects human health [8]. For example, Cadmium (Cd) can cause prostatic hyperplasia, lung cancer and other chronic diseases. In addition, even elements necessary for health may be harmful in high levels [9], such as zinc, which plays a critical role in the development of the nervous system and Intelligence Quotient (IQ) [10]. Generally, exposure to heavy metals can lead to inflammation, skin irritation, immune system damage and cancer risk [11].

The impact of human activities causes re-distribution of heavy metals in a specific space. The types of pollution sources can be preliminarily judged and screened via analyzing the distribution pattern of heavy metal pollution [12]. In the analysis of pollution sources, multivariate statistical analysis is mainly conducted through analyzing the data to find out its internal laws, and, then, finding the main pollution factors. In addition, combined with GIS technology, the spatial interpolation of soil heavy metal content can be used to describe the two-dimensional and three-dimensional distribution of heavy metals, so that the research results can be more accurately visualized [13]. Currently, soil ecological risk assessment methods mainly include the index method, quotient method, fuzzy comprehensive evaluation, geoaccumulation index, potential ecological risk index and pollution load index [14]. These evaluation methods can directly reflect the spatial changing trend and pollution level of soil pollutants, and, thus, are used to qualitatively and quantitatively assess soil ecological and environmental risks, thereby effectively solving practical problems in soil ecological risk assessment. There are various types of urban environmental pollution sources and complex risk exposure pathways [15]. Multiple methods can be used in a comprehensive manner.

Compared with other cities or rural areas, in addition to pollution from industry and agriculture, Tianjin is also affected by factors such as seawater erosion and port construction. Since the middle of the 20th century, Tianjin’s suburban farmland has been irrigated with sewage for a long time, making Tianjin one of the five largest sewage irrigation areas in China. Additionally, with the development of industry and the expansion of the city, heavy metal pollution in Tianjin’s soil has become increasingly serious. Therefore, it is of great importance to reveal the characteristics of heavy metal pollution in different types of land in Tianjin.

Based on the above considerations, this study took Tianjin as the research area, soil samples from different land use types were collected, and the content of heavy metals Cd, Pb, Cu, Zn, Cr, As, Ni in soils were characterized. By using GIS spatial analysis technology with geostatistical technology to perform Kriging interpolation, combined with multivariate statistical methods, the main sources of soil heavy metal accumulation and distribution in Tianjin were analyzed. Using the combination of geoaccumulation index, Nemerow comprehensive pollution index, and pollution load index, soil pollution levels of heavy metals were comprehensively evaluated.

## 2. Materials and Methods

### 2.1. Sample Collection and Analysis

**Figure 1:**
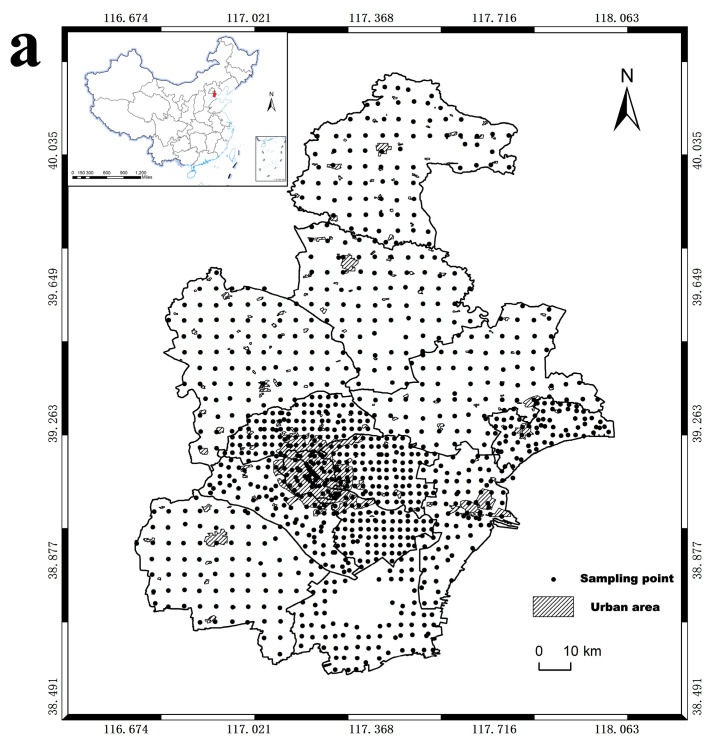

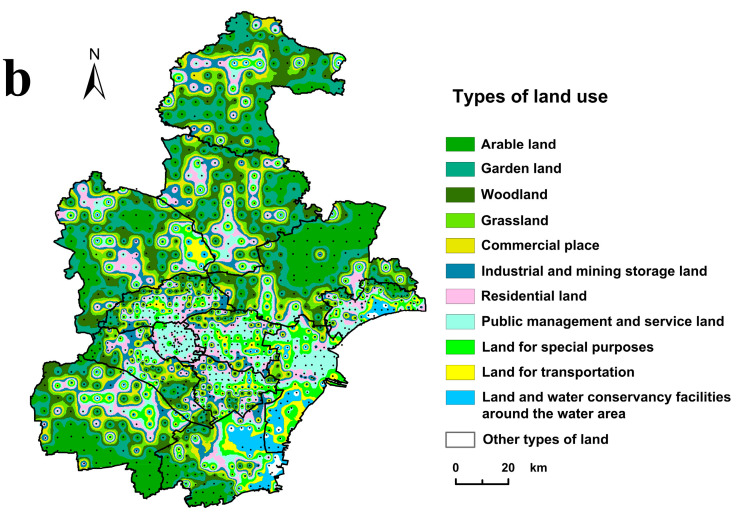
Sampling map of soil samples in Tianjin in this study. (**a**) Map of soil sampling location; (**b**) Distribution map of land use types at sampling points.

The land area in the outer suburbs is relatively large, about 3–4 km per grid, and the land area in the suburbs is small, about 1–2 km per grid. There is one monitoring point in each grid. During soil sampling, areas with poor micro-domain geomorphology were avoided, and samples in typical plots in each grid were collected. A total of 1031 soil samples were obtained (Figure 1a). According to the national land classification standards (GB/T 21010-2017 and GB 50137-2016), the collected soil samples came from 12 different types of soil (Table S1). Land use types were filled with different colors according to the soil classifications, and the details are shown in Figure 1b.

After removing the top soil, soils with a depth of 0–20 cm were collected. Soil was mixed from 5 sampling points, based on the “Plum Blossom Sampling Method”. Each soil sample was obtained about 1.5 kg by the quarter method and placed in a double-layer plastic bag, and the field sampling information was recorded. After the soil collection, all samples were air-dried in a greenhouse, and plant residues, stones and other debris were removed. All samples were crushed with a wooden hammer and passed through a 100-mesh nylon sieve, and then stored in plastic bags for later use. Taking the sieved soil sample, after HNO_3_-HF combined digestion, we used an inductively coupled plasma mass spectrometer (Agilent Technologies 7900 ICP-MS) to determine the concentration of Cu, Zn, As, Pb, Cr, Ni, Cd. The experimental water was ultrapure water (18.2 MΩ). National soil standard samples (GSS-8, GSS-23 and GSS-27) were added during the analysis process for quality control. The recovery rate of standard samples was between 87% and 112%, and the precision (RSD) was between 0.22% and 1.22%. The pH value of soil samples was measured by the potentiometric method, from the extraction of deionized water and soil (water-soil ratio 1:1) [16].

### 2.2. Evaluation Method of Soil Heavy Metals

#### 2.2.1. Geoaccumulation Index Method

The geoaccumulation index method was used to evaluate the level of heavy metal pollution in sediments, and is now widely used to evaluate soil heavy metal pollution [17]. This method uses the geochemical background value in the soil as the standard, and the calculation formula is:

(1) $$I_{\mathrm{geo}}=\log_{2}\left(\frac{C_{i}}{1.5B_{i}}\right)$$

In the formula, Igeo is the geoaccumulation index of heavy metals; *Ci* is the measured value of heavy metal *i* (mg/kg); *Bi* is the geochemical background value of heavy metal *i* (mg/kg). This study used the soil background value of Tianjin as the evaluation standard (Table S2); 1.5 was the compensation coefficient, which represented natural fluctuations and slight human input caused by diagenetic effects. The geoaccumulative index can be divided into 7 levels (Table S3).

#### 2.2.2. Nemeiro Composite Index Method

On the basis of the single factor index, the Nemeiro comprehensive index method takes into account the average value and the highest value of the single factor pollution index, and highlights the impact of the most polluted pollutants on environmental quality [18]. The calculation formula of Nemeiro composite index method is:

(2) $$P_{sum}=\sqrt{\frac{{\left(\frac{1}{n}\sum_{n=1}^{n}P_{i}\right)}^{2}+{\left[\max(P_{i})\right]}^{2}}{2}}$$

In the formula, *P_i_* is the single factor pollution index of heavy metal *i* in the soil; *C_i_* is the measured value of heavy metal *i* (mg/kg); *S_i_* is the evaluation standard value of heavy metal *i* (mg/kg). Soil pollution risk control standard (for trial implementation)” (GB15618-2018) and “Soil pollution risk control standard for construction land for soil environmental quality (for trial implementation)” (GB36600-2018) were used as the evaluation criteria (Table S2); *P_sum_* is the Nemerow comprehensive pollution index; max (*P_i_*) is the maximum value of the single-factor pollution index of each heavy metal element in the soil; *n* is the number of heavy metal species measured in the soil, and *n* = 7 in this study. The index *P* was divided into the same level in the single factor index method and Nemeiro composite index method (Table S4).

#### 2.2.3. Pollution Load Index

The pollution load index method is also an evaluation method that combines single factor index and comprehensive evaluation [19]. It can be used to evaluate the overall pollution status of soil heavy metals. It is widely used in the evaluation of air, water and soil pollution. Its formula is:

(3) $$CF_{i}=\frac{C_{i}}{B_{i}}$$

(4) $$PLI=\sqrt[{n}]{CF_{1}\times CF_{2}\times L\times CF_{n}}$$

In the formula, *Ci* and *Bi* are the same as Equation (1); PLI is the heavy metal pollution load index; *n* is the number of heavy metals to be evaluated, and *n* = 7 in this study. The value of PLI was divided into 4 levels (Table S5).

## 3. Results and Discussion

### 3.1. Spatial Distribution Characteristics of Soil Heavy Metals in Different Land Use Types in Tianjin

As shown in Table 1, the median contents of the seven heavy metal elements in the soil of Tianjin were Cr (70.7 mg/kg), Ni (27.9 mg/kg), Cu (24.3 mg/kg), Zn (79.1 mg/kg), As (11.9 mg/kg), Pb (24.3 mg/kg), and Cd (0.15 mg/kg). The element content of Cr, Ni, Cu was lower than the background value. The element content of Zn and Pb was close to the background value, and the element content of As and Cd was higher than the background value. Compared with the study of [20], the geometric means of Cr, Ni and Zn in urban soils of our research were higher than that of Beijing. In addition, in comparison with the heavy metal contents of agricultural soils in Shanghai area by [21], the contents of Cd, Cr and Pb in this study were relatively low, while the contents of As were relatively high. High-quality cultivated land distributed in suburban regions is strongly affected by human activities, which has resulted in high heavy metal contents. The As concentration was significantly higher in soils of a moderate quality level. Moreover, although nonsignificant, the Cd content was higher in moderate-quality cultivated land. In recent years, due to the requirement of cultivated land quality improvement, moderate-quality farmland must be upgraded to a high quality level, which could lead to the intensive application of fertilizers and pesticides to raise the quality of arable land and agricultural production, and Cd and As are usually present in fertilizers, especially phosphate fertilizers and pesticides [22].

**Figure 2:**
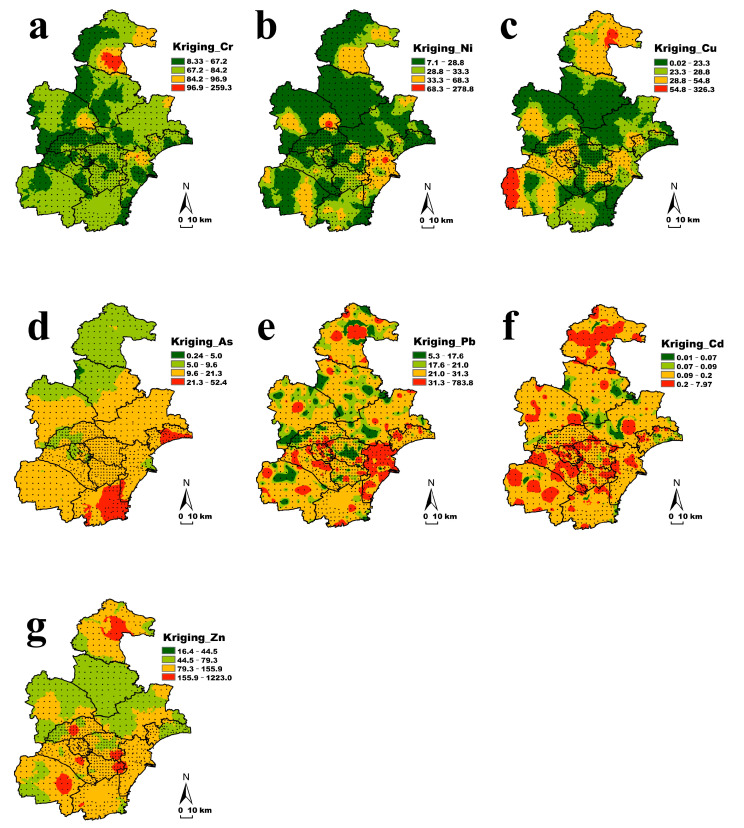
Spatial distributions of seven heavy metals ((**a**): Cr; (**b**): Ni; (**c**): Cu; (**d**): As; (**e**): Pb; (**f**): Cd and (**g**): Zn) in soils of Tianjin.

Figure 2 shows the spatial distribution of seven heavy metals in the soil of the study area. The Cr content at most points was lower than the background value of Tianjin soil. In the east, especially the southeast, Cr had a high accumulation index. In the east of Wuqing District, the north of Tanggu District and the northeast corner of Ninghe District, the value of Cr was relatively higher. The green filled area of Ni and Cu was relatively large, and the content in most areas of Tianjin was lower than the background value. The content of Ni over background value was more scattered, while the yellow area of Cu was connected together, showing obvious non-point source pollution. Furthermore, especially in Jizhou District, almost all the points of Cu content exceeded the background value, and there was also a cluster of over-standard points at the junction of southern Jinghai District and Hebei Province. The accumulation of Zn and As in the study area was slightly higher, and there was almost no light green area. The accumulation of Zn in Baodi District was below the background value, and there were accumulations of over-standard points in other jurisdictions of Tianjin. The coefficient of variation of the As element data was small. The yellow patches in the figure were connected in a large area, and the content showed obvious characteristics of being high in the south and low in the north. The accumulation value was the highest in the Binhai New Area. The Pb and Cd content data showed strong variations. It can be seen from Figure 2 that they were characterized by obvious point source pollution. The accumulation of the two in the soil of the study area was likely to be related to man-made production and living activities.

All soil samples collected in the study area were classified according to land use types, and the statistical results of the accumulation of heavy metal content are shown in Table 2. It can be seen from Table 2 that the element content of Ni element in the soil was lower than the background value under all land use types, and the value was higher under the four types of farm land, garden, woodland and grassland and special land types, exceeding 70 mg/kg. The lowest content was in the type of commercial drug (only 61.3 mg/kg). The content of Ni in grassland was significantly higher than other land use types (34.5 mg/kg), and the coefficient of variation was higher in industrial and mining storage land.

The Cu content was the highest in grassland (28.6 mg/kg), which was close to the background value, and the coefficient of variation was extremely high (over 2) in commercial and transportation land. The content of Zn in arable land, garden land, woodland, residential land, and public management and public service land all exceeded the standard value, especially the highest, which was in grassland (107.6 mg/kg). Although the content of Zn in industrial and mining storage land and transportation did not exceed the standard, it showed extremely high coefficient of variation, indicating that Zn in these two land use types was significantly affected by the specific location.

Except for residential land, the content of As in the soil of other land use types was higher than the background value, and the highest was in grassland (20.5 mg/kg). The content of Pb and Cd in all land use types exceeded the background value. The content of Pb in special land types was slightly lower (21.3 mg/kg), and the coefficient of variation of the content in garden soil was extremely high (4.0). The coefficient of variation of the Cd element in gardens was as high as 5.6, and it was also extremely high in the soil of industrial and mining storage land, residential land and land and water conservancy facilities around the water area.

In general, among the 12 land use types, the seven heavy metals in the grassland soil all exceeded the background value, and the content values of Zn and As were much higher than the content levels of other land use types. The coefficient values of each element were low, indicating that the cumulative distribution of heavy metals in grassland was relatively even. The coefficients of variation of metals in garden land, industrial and mining storage land, residential land and land for transportation were also higher than that of other land use types. It has been reported that heavy metals with relatively wide concentration ranges and high coefficients of variation mean that anthropogenic sources are present [23].

### 3.2. Evaluation of Soil Heavy Metal Pollution in Tianjin

#### 3.2.1. Evaluation of Geoaccumulation Index

The average geoaccumulation index of seven heavy metals in Tianjin soil was Cu (−0.90) < Cr (−0.89) < Ni (−0.86) < Zn (−0.53) < Pb (−0.36) = As (−0.36) < 0 < Cd (0.15) (Table 3). Except for Cd, the other heavy metal elements were at the pollution-free level, and Cd was pollution-free to moderate pollution level. From the view of different jurisdictions, the geoaccumulation index of As in Binhai New Area (Tanggu, Hangu, Dagang) was pollution-free to moderate pollution level (0.22), and in the urban and suburban districts (Dongli, Xiqing, Beichen and Jinnan) the accumulation index of Cd element was higher than that of other jurisdictions. From the view of different types of land use, As was at a pollution-free to moderate pollution level in waters and water conservancy facilities, Cd was at a pollution-free accumulation level in industrial and mining storage land, water areas and water conservancy, and the accumulation level in commercial areas was also low (0.00), The accumulation index of garden land, residential land and land for transportation was higher than that of other land use types, which can be considered due to the addition of new man-made pollution sources.

#### 3.2.2. Evaluation of Nemeiro Comprehensive Pollution Index

The single-factor pollution index of the seven heavy metal elements was calculated by the single-factor pollution index method, and then the Nemeiro comprehensive pollution index of the sample was calculated. According to the results of the Nemeiro comprehensive index in Table 4, the level of heavy metal pollution in the soil in Tianjin was relatively low, and the pollution levels in the soil in different jurisdictions and different land use types in Tianjin were all low. The Nemeiro composite index of Binhai New Area was close to the warning limit (0.7), and the Nemeiro composite index of land use types for industrial, mining, storage and transportation land was close to the warning limit.

**Figure 3:**
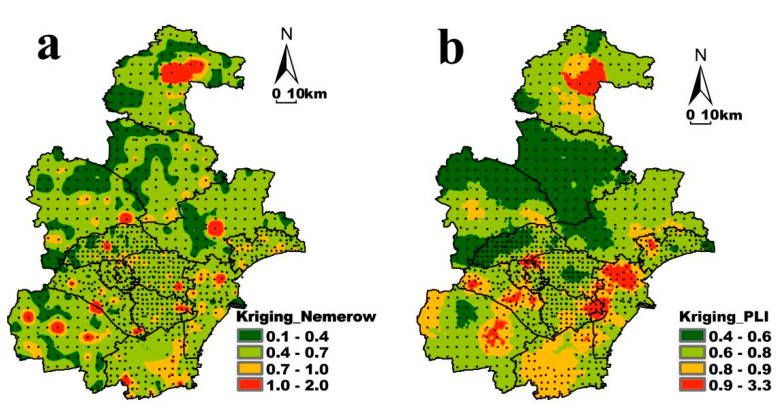
Nemerow comprehensive evaluation index (**a**) and Pollution load index (**b**) of heavy metals in soil.

In order to visually find out the points where the Nemeiro Comprehensive Pollution Index exceeds the warning limit from the overall data and reach light, or even severe, pollution, ESRI ArcMap 13.0 was used to interpolate the Nemeiro Comprehensive Pollution Index value of the sampling points, and the results are shown in Figure 3a. The high pollution index points were scattered in the study area, but the points where the pollution level exceeded the warning limit were gathered in the central part of Jizhou District and the southeastern part of Dagang District, showing the characteristics of non-point source pollution. Combined with the sampling point table, the high value points of the comprehensive pollution index were: the urban area of Jizhou District, east of Yuqiao Reservoir; the cultivated land of Lingtou Village in Ninghe District; Shangmatai Town Industrial Zone in Wuqing District; Danjiang Road in Beichen District With Huaidong Road Industrial Zone; Dongli District Junyi Industrial Park; Duliujian River on the north bank of Tuanbowa Reservoir in Jinghai District; and cultivated land in Yangkezhuang Village, Xiqing District.

#### 3.2.3. Pollution Load Index Evaluation

The heavy metal pollution load index of Tianjin was 0.73, which was a pollution-free level (Table 5). The pollution load index was calculated according to different jurisdictions and different land use types. The PLI value of each jurisdiction and land use type had little difference, and they were all at the pollution-free level, which was consistent with Nemeiro’s comprehensive evaluation index. The PLI value of Binhai New Area was slightly higher (0.80), and the PLI value of the five outer suburbs (Jizhou, Wuqing, Ninghe, Jinghai, and Baodi) was slightly lower (0.68).

The areas with higher soil heavy metal pollution load index in Tianjin were mainly in the central part of Jizhou, the southern part of Jinghai District, the northern part of Tanggu District, the central part of Jinnan District and the northern part of the urban area (Figure 3b). The pollution load index of most areas in the northern area of Wuqing and Baodi was very low. In general, the interpolation results of the pollution load index value and the Nemeiro comprehensive evaluation index value were consistent. Combining the sampling points and satellite image, the high pollution load index points, or areas, were: the south bank of Yuqiao Reservoir in Jizhou District; Yunjingdao Industrial Park, Shangmatai Town, Wuqing District; two industrial zones in Dongli District; Jinghai District Ziya River and Daqiuzhuang town residential area; Tuanbo Express Road in Xiqing District (Along the Chentaizi Drainage River); Tanggu District Yangbei Logistics Park and Babao Village urban residential area; Jinnan District G319 Jinjin Expressway (Dagu sewage discharge on the north bank of Hebei); urban area (near Tianjin North Station).

### 3.3. Sources of Soil Heavy Metals in Tianjin

Spearman correlation analysis was performed on seven soil heavy metals in Tianjin, and the results are shown in Table 6. Except for Cd and As, the heavy metal elements in the soil of Tianjin had significant correlations in pairs. Among them, the significant correlation level of Cu and As was *p* < 0.05, and the correlation levels of the other elements all reached *p* < 0.01.

Considering that there may be differences in the accumulation of heavy metal elements in the soils of different regions, a correlation analysis of heavy metal elements in the soil of each jurisdiction was further done, and the results are shown in Table 7. The significant correlation between the seven heavy metals was roughly the same in different jurisdictions of Tianjin, but the correlation strength was different. Cr-Ni had a significant correlation in all jurisdictions, and a strong and significant correlation in Baodi, Ninghe, Jizhou and the urban area; Cr-Pb had a significant correlation in all jurisdictions, and a strong and significant correlation in Wuqing; Ni-Cu, Ni-Zn, Ni-Pb, and Ni-Cr had significant correlations in most jurisdictions, and Ni-Cu and Ni-Pb were mostly highly correlated; Cu-Ni, Cu-Cr, Cu-Zn, Cu-Pb had a significant correlation in most jurisdictions and Cu-Ni, Cu-Zn, Cu-Pb showed a strong and significant correlation; Cu-Cd showed a highly significant correlation in Dongli, Beichen, Ninghe and urban areas; Zn-Ni, Zn-Cu, Zn-Pb, and Zn-Cd had significant correlations in most jurisdictions, among which Zn-Cd had a strong and significant correlation in all jurisdictions; As and other elements were weakly correlated or uncorrelated in all jurisdictions. As-Zn and As-Pb showed weak negative correlation in Binhai New Area, and As-Cd showed weak negative correlation in Beichen, Baodi, Jizhou and Binhai New Area; Cd -Zn and Cd-Pb showed a strong and significant correlation in most jurisdictions, and the degree of significant correlation in each jurisdiction was relatively consistent.

Generally, the correlation between Cr and other metal elements was relatively general in the four suburbs (Dongli, Xiqing, Jinnan, Beichen), and strong in the five outer suburbs (Beichen, Wuqing, Baodi, Jinghai and Jizhou), the strongest was in the urban area. There was a correlation between heavy metal elements in urban soils (except As), the correlation between heavy metal elements in the soil of Ninghe was also strong (except As, Cr), and the correlation between heavy metal elements in the soil of Jinnan was relatively not obvious.

**Figure 4:**
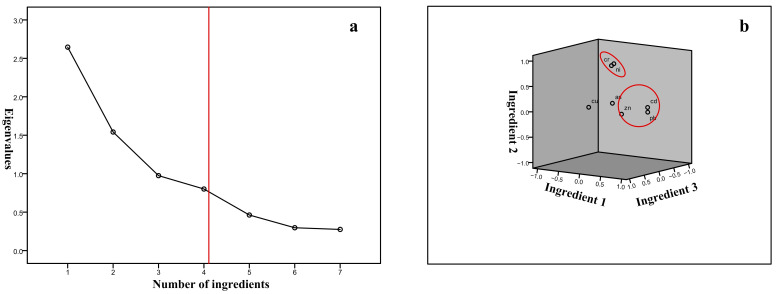
Gravel map of principal component analysis (**a**) and component map of rotating space (**b**).

Principal component analysis was performed on the contents of seven heavy metal elements in 1031 soil samples. The results of the principal component analysis are shown in Table S6. The component matrices and rotating component matrices of the 4 principal components extracted by the principal component analysis are shown in Table S7. The principal component analysis gravel diagram and the component diagram in the rotating space are shown in Figure 4. In this study, the components with eigenvalues greater than 0.8 were selected as the principal components, and a total of 4 principal components were extracted, which could explain 85.2% of the variance in total.

PC1 mainly reflects the composition information of Zn, Pb, and Cd, explaining 37.799% of the variance variable. These three heavy metals showed strong correlations in most soils in Tianjin (Figure 4 and Table 7). In nature, Cd is an associated element of lead-zinc ore and copper-lead-zinc ore [24]. The main human factors of Cd accumulation include: lead-zinc ore mining, three waste emissions in the smelting process, coal and crude oil combustion, and cadmium-containing fertilizers in agricultural production activities and so on [25]. Pb in the soil mainly comes from the three wastes discharged from lead mine mining and atmospheric deposition from vehicle exhaust emissions [26]. The accumulation of Zn in the soil is not only affected by the soil parent material, but also mainly due to emissions from industrial production [27]. In addition, Tianjin has a long history of sewage irrigation. The sewage irrigation area of Beijing Sewage River in Wuqing District, Dagu Sewage River Sewage Irrigation District in Xiqing, and Beitang Sewage River Sewage Irrigation District in Dongli, all exerted the effect of accumulation of Cd, Pb, Zn, Cu and As [28]. Cd, Pb and Zn in the study area, all exceeding the background value, and the variability of the data was strong to extremely strong. Therefore, it was speculated that the sources of these three elements in the study were mainly derived from man-made activities, such as industrial production and transportation. This was consistent with a previous study [29]. PC2 mainly reflected the composition information of Cr and Ni, explaining the information with a variance of 22.03%. Both of these two elements are diagenetic elements. The average content in the study was lower than the background value and the coefficient of variation was small, so PC2 was the source of soil parent material. PC3 mainly reflected the composition information of Cu. The explained variance accounted for 13.93%. It was obvious from the distribution of Cu in Tianjin that the points with high Cu content were clustered in the east and the east of Jizhou District. In the west of Jinghai District, Tianjin’s main metal and non-metal mineral resources were concentrated in the Jizhou mountain area, while in the west of Jinghai District, there were many non-ferrous metal smelting and processing plants, such as copper mills, copper demolition plants, copper rice plants, and scrap metal processing plants. Therefore, it was inferred that PC3 was a mining and metallurgical source. PC4 mainly reflected the composition information of As, and the explained variance accounted for 11.443%. The accumulation characteristics of As in the study were quite special. Its correlation with other metal elements was low. In the study area, the pollution level of As was low in the north but high in the south. The highest concentration points were concentrated in the Binhai New Area. As is also a diagenetic element, and its accumulation in the soil comes from both natural and anthropogenic sources. The anthropogenic sources were mainly the application of agrochemical products, mining, and industry. Studies have shown that the spatial distribution of As in Tianjin’s soil was related to the distribution of arsenic emission enterprises in Tianjin and the irrigation of farmland [30].

## 4. Conclusions

In this paper, we collected the surface soil in Tianjin at high density, combined with land use types, and used multiple data analysis and multivariate statistics to study the distribution characteristics and pollution sources of heavy metals in soil, and evaluated the pollution levels by various indices. The results showed that the accumulation of heavy metals was highest in grassland, and the coefficient of variation of heavy metal content was higher in garden land, industrial and mining storage land, residential land and transportation land, indicating that the soil heavy metal content under these land use types was more obviously disturbed by human factors. The evaluation results of the ground accumulation index method show that the soil in Tianjin is free of pollution except for Cd which is free of pollution to moderate pollution level. Cd, Pb and Zn are the main contributors to pollution by anthropogenic factors, such as traffic and industry; Cr and Ni are the main contributors to pollution by soil parent material; Cu is the main contributor to pollution by mining and metal smelting; As is presumed to be the main contributor by agriculture and surface runoff. The results can provide reference significance for agricultural environmental protection in urban development.

## Figures and Tables

**Table 1:** Statistic of heavy metal concentrations in the soil of Tianjin (mg/kg).

Project	Cr	Ni	Cu	Zn	As	Pb	Cd
Minimum value	8.33	7.1	0.02	16.4	0.24	5.3	0.015
Maximum value	259.3	278.8	326.3	1223.0	52.4	783.8	7.97
Arithmetic mean	70.7	29.4	27.5	94.7	13.3	27.1	0.20
Geometric mean	68.1	27.6	23.2	82.3	11.2	24.5	0.15
25th percentile	59.3	23.1	17.8	63.1	8.0	20.1	0.11
50th percentile	70.7	27.9	24.3	79.1	11.9	24.3	0.15
75th percentile	79.9	33.4	30.9	101.4	17.2	29.3	0.20
90th percentile	89.3	38.8	42.8	136.0	22.4	36.6	0.28
Standard deviation	19.5	16.3	20.3	80.9	7.4	29.0	0.34
Coefficient of variation	0.28	0.55	0.74	0.85	0.56	1.07	1.70

**Table 2:** Statistic of heavy metals centent in soils of different land use types in Tianjin (mg/kg).

Land Use Type	Project	Cr	Ni	Cu	Zn	As	Pb	Cd
Number								
1	content	73.8 ± 17.2	28.6 ± 7.8	25.6 ± 19.7	80.8 ± 49.9	11.2 ± 6.6	24.5 ± 10.1	0.15 ± 0.24
*n* = 294	CV	0.2	0.3	0.8	0.6	0.6	0.4	1.6
2	content	73.2 ± 18.9	27.6 ± 7.5	26.2 ± 17.2	86.9 ± 116.1	10.1 ± 5.8	24.1 ± 96.9	0.18 ± 1.00
*n* = 62	CV	0.3	0.3	0.7	1.3	0.6	4.0	5.6
3	content	70.7 ± 19.4	29.3 ± 9.3	25.2 ± 13.7	76.2 ± 54.2	11.0 ± 6.0	24.6 ± 10.4	0.15 ± 0.22
*n* = 94	CV	0.3	0.3	0.5	0.7	0.5	0.4	1.4
4	content	78.4 ± 17.1	34.5 ± 7.7	28.6 ± 8.4	107.6 ± 96.4	20.5 ± 9.6	27.1 ± 5.5	0.17 ± 0.10
*n* = 10	CV	0.2	0.2	0.3	0.9	0.5	0.2	0.6
5	content	61.3 ± 18.0	27.0 ± 7.4	25.2 ± 53.5	75.0 ± 24.7	10.7 ± 1.8	23.3 ± 12.3	0.16 ± 0.05
*n* = 7	CV	0.3	0.3	2.1	0.3	0.2	0.5	0.3
6	content	65.7 ± 30.9	26.0 ± 36.2	20.1 ± 22.7	68.7 ± 147.8	14.9 ± 8.5	22.8 ± 26.5	0.13 ± 0.37
*n* = 51	CV	0.5	1.4	1.1	2.2	0.6	1.2	2.9
7	content	66.3 ± 18.7	26.0 ± 13.8	25.2 ± 27.1	90.1 ± 93.9	8.6 ± 6.1	26.1 ± 52.1	0.16 ± 0.34
*n* = 59	CV	0.3	0.5	1.1	1.0	0.7	2.0	2.1
8	content	69.6 ± 20.9	29.4 ± 23.2	26.0 ± 12.4	82.6 ± 41.9	10.9 ± 5.7	25.4 ± 11.1	0.15 ± 0.23
*n* = 209	CV	0.3	0.8	0.5	0.5	0.5	0.4	1.5
9	content	73.6 ± 5.4	28.5 ± 3.5	27.3 ± 16.8	71.8 ± 18.9	14.3 ± 6.4	21.3 ± 8.4	0.10 ± 0.04
*n* = 5	CV	0.1	0.1	0.6	0.3	0.5	0.4	0.4
10	content	69.3 ± 17.8	28.2 ± 7.8	22.9 ± 46.2	76.5 ± 193.9	10.2 ± 8.4	22.9 ± 8.9	0.15 ± 0.25
*n* = 48	CV	0.3	0.3	2.0	2.5	0.8	0.4	1.7
11	content	67.7 ± 18.8	26.8 ± 9.7	20.3 ± 18.2	75.3 ± 59.3	17.4 ± 7.9	23.6 ± 7.9	0.11 ± 0.28
*n* = 116	CV	0.3	0.4	0.9	0.8	0.5	0.3	2.5
12	content	69.7 ± 17.9	26.0 ± 22.7	20.0 ± 11.5	69.4 ± 85.1	16.0 ± 9.7	22.9 ± 9.0	0.13 ± 0.06
*n* = 76	CV	0.3	0.9	0.6	1.2	0.6	0.4	0.5

**Table 3:** Geoaccumulation index of heavy metals in soils of Tianjin.

Classification	Num	Cr	Ni	Cu	Zn	As	Pb	Cd
Tianjin	1031	−0.89 ± 0.41	−0.86 ± 0.48	−0.90 ± 0.90	−0.53 ± 0.67	−0.36 ± 0.92	−0.36 ± 0.54	0.15 ± 0.86
Four suburban districts	339	−0.92 ± 0.37	−0.88 ± 0.47	−0.97 ± 1.10	−0.47 ± 0.77	−0.31 ± 0.71	−0.43 ± 0.53	0.30 ± 0.87
Outer suburb five districts	397	−0.86 ± 0.41	−0.92 ± 0.49	−0.92 ± 0.88	−0.68 ± 0.64	−0.64 ± 0.97	−0.48 ± 0.51	0.02 ± 0.93
Binhai New Area	214	−0.86 ± 0.49	−0.72 ± 0.48	−0.85 ± 0.58	−0.41 ± 0.51	0.22 ± 0.87	−0.12 ± 0.46	0.07 ± 0.71
Urban district	81	−0.98 ± 0.35	−0.83 ± 0.36	−0.60 ± 0.66	−0.35 ± 0.65	−0.77 ± 0.79	−0.16 ± 0.70	0.39 ± 0.67
Cultivated land	294	−0.83 ± 0.36	−0.85 ± 0.42	−0.81 ± 0.82	−0.55 ± 0.58	−0.48 ± 0.91	−0.38 ± 0.43	0.20 ± 0.82
Garden plot	62	−0.86 ± 0.42	−0.89 ± 0.43	−0.68 ± 0.77	−0.47 ± 0.76	−0.68 ± 1.03	−0.31 ± 0.82	0.34 ± 1.05
Woodland	94	−0.85 ± 0.39	−0.80 ± 0.46	−0.85 ± 0.77	−0.60 ± 0.57	−0.43 ± 0.78	−0.39 ± 0.44	0.17 ± 0.86
Place of commercial use	7	−1.12 ± 0.46	−0.96 ± 0.41	−0.62 ± 1.37	−0.67 ± 0.49	−0.40 ± 0.25	−0.41 ± 0.61	0.00 ± 0.55
Industrial and mining storage	51	−1.09 ± 0.69	−0.95 ± 0.65	−1.18 ± 1.11	−0.53 ± 0.90	−0.15 ± 0.92	−0.41 ± 0.75	−0.02 ± 1.05
Residential land	59	−0.97 ± 0.42	−0.90 ± 0.52	−0.68 ± 0.88	−0.27 ± 0.82	−0.70 ± 0.84	−0.21 ± 0.76	0.34 ± 0.90
Public service land	209	−0.88 ± 0.37	−0.79 ± 0.52	−0.85 ± 0.68	−0.51 ± 0.60	−0.43 ± 0.81	−0.29 ± 0.53	0.23 ± 0.77
Traffic land	48	−1.00 ± 0.48	−0.91 ± 0.42	−0.93 ± 0.98	−0.38 ± 0.92	−0.73 ± 1.28	−0.40 ± 0.47	0.35 ± 0.90
Water area land	116	−0.93 ± 0.39	−0.93 ± 0.48	−1.12 ± 0.76	−0.65 ± 0.67	0.14 ± 0.79	−0.45 ± 0.50	−0.15 ± 0.94

**Table 4:** Single factor pollution index and comprehensive pollution index of soil heavy metals.

Classification	*P_i_*							*P_sum_*	Class of Pollution
	Cr	Ni	Cu	Zn	As	Pb	Cd		
Tianjin	0.52	0.11	0.16	0.32	0.42	0.11	0.19	0.56	Security
Four suburban districts	0.56	0.10	0.14	0.35	0.38	0.09	0.17	0.57	Security
Outer suburb five districts	0.42	0.13	0.21	0.28	0.40	0.12	0.26	0.51	Security
Binhai New Area	0.55	0.11	0.14	0.32	0.59	0.11	0.16	0.63	Security
Urban district	0.78	0.06	0.004	0.35	0.20	0.05	0.004	0.58	Security
Cultivated land	0.29	0.15	0.29	0.30	0.49	0.15	0.32	0.47	Security
Garden plot	0.29	0.15	0.31	0.35	0.44	0.22	0.52	0.57	Security
Woodland	0.29	0.16	0.27	0.29	0.49	0.15	0.32	0.46	Security
Place of commercial use	0.72	0.03	0.002	0.26	0.18	0.03	0.002	0.53	Security
Industrial and mining storage	0.78	0.03	0.001	0.37	0.26	0.04	0.003	0.62	Security
Residential land	0.80	0.19	0.02	0.40	0.52	0.09	0.01	0.64	Security
Public service land	0.84	0.04	0.001	0.30	0.20	0.03	0.003	0.61	Security
Traffic land	0.78	0.03	0.002	0.43	0.20	0.03	0.003	0.66	Security
Water area land	0.27	0.15	0.23	0.29	0.72	0.14	0.28	0.60	Security

**Table 5:** Results and comparison of pollution load index and Nemerow comprehensive evaluation index.

Classification	Num	Pollution Load Index		Nemero Comprehensive Evaluation Index	
		PLI	Class of Pollution	*P_sum_*	Class of Pollution
Tianjin	1031	0.73 ± 0.25	Pollution-free	0.56	Security
Four suburban districts	339	0.74 ± 0.26	Pollution-free	0.57	Security
Outer suburb five districts	397	0.68 ± 0.25	Pollution-free	0.51	Security
Binhai New Area	214	0.80 ± 0.22	Pollution-free	0.63	Security
Urban district	81	0.76 ± 0.26	Pollution-free	0.58	Security
Cultivated land	294	0.73 ± 0.22	Pollution-free	0.47	Security
Garden plot	62	0.77 ± 0.40	Pollution-free	0.57	Security
Woodland	94	0.72 ± 0.23	Pollution-free	0.46	Security
Place of commercial use	7	0.70 ± 0.28	Pollution-free	0.53	Security
Industrial and mining storage	51	0.72 ± 0.36	Pollution-free	0.62	Security
Residential land	59	0.76 ± 0.28	Pollution-free	0.64	Security
Public service land	209	0.74 ± 0.24	Pollution-free	0.61	Security
Traffic land	48	0.71 ± 0.27	Pollution-free	0.66	Security
Water area land	116	0.70 ± 0.23	Pollution-free	0.60	Security

**Table 6:** Spearman correlation analysis of seven heavy metals in soils of Tianjin.

	Cr	Ni	Cu	Zn	As	Pb	Cd
Cr	1.000						
Ni	0.762 **	1.000					
Cu	0.562 **	0.790 **	1.000				
Zn	0.533 **	0.726 **	0.797 **	1.000			
As	0.325 **	0.299 **	0.071 *	0.095 **	1.000		
Pb	0.549 **	0.722 **	0.761 **	0.824 **	0.181 **	1.000	
Cd	0.443 **	0.615 **	0.713 **	0.786 **	−0.052	0.688 **	1.000

Note: *, *p <* 0.05; **, *p <* 0.01.

**Table 7:** Spearman correlation analysis of seven heavy metals in soils of different districts of Tianjin.

Element	DL *n* = 93	XQ *n* = 72	JN *n* = 78	BC *n* = 96	WQ *n* = 82	BD *n* = 76	JH *n* = 78	NH *n* = 79	JZ *n* = 82	UD *n* = 81	BH *n* = 214
Cr-Ni	0.680 **	0.828 **	0.580 **	0.697 **	0.638 **	0.955 **	0.756 **	0.854 **	0.895 **	0.914 **	0.805 **
Cr-Cu	0.555 **	0.693 **	0.153	0.566 **	0.648 **	0.890 **	0.454 **	0.710 **	0.493 **	0.792 **	0.791 **
Cr-Zn	0.514 **	0.569 **	0.242 *	0.492 **	0.826 **	0.774 **	0.639 **	0.680 **	0.385 **	0.722 **	0.624 **
Cr-As	0.443 **	0.390 **	0.333 **	0.521 **	0.086	0.427 **	0.387 **	0.377 **	0.079	0.570 **	0.158 *
Cr-Pb	0.577 **	0.567 **	0.308 **	0.633 **	0.862 **	0.775 **	0.641 **	0.822 **	0.274 *	0.814 **	0.623 **
Cr-Cd	0.425 **	0.428 **	0.166	0.465 **	0.659 **	0.435 **	0.601 **	0.617 **	0.092	0.732 **	0.616 **
Ni-Cu	0.855 **	0.777 **	0.625 **	0.863 **	0.903 **	0.891 **	0.779 **	0.902 **	0.599 **	0.884 **	0.833 **
Ni-Zn	0.751 **	0.622 **	0.600 **	0.720 **	0.794 **	0.764 **	0.721 **	0.830 **	0.507 **	0.797 **	0.756 **
Ni-As	0.458 **	0.353 **	0.135	0.428 **	0.379 **	0.488 **	0.293 **	0.358 **	0.257 *	0.485 **	−0.046
Ni-Pb	0.707 **	0.641 **	0.782 **	0.761 **	0.803 **	0.742 **	0.776 **	0.877 **	0.344 **	0.787 **	0.805 **
Ni-Cd	0.582 **	0.446 **	0.584 **	0.676 **	0.600 **	0.401 **	0.697 **	0.651 **	0.191	0.815 **	0.680 **
Cu-Zn	0.913 **	0.689 **	0.724 **	0.823 **	0.861 **	0.899 **	0.611 **	0.944 **	0.813 **	0.904 **	0.731 **
Cu-As	0.285 **	0.214	−0.027	0.200	0.260 *	0.190	0.142	0.270 *	−0.103	0.252 *	−0.092
Cu-Pb	0.878 **	0.715 **	0.656 **	0.781 **	0.818 **	0.895 **	0.838 **	0.907 **	0.567 **	0.862 **	0.711 **
Cu-Cd	0.742 **	0.622 **	0.621 **	0.776 **	0.655 **	0.656 **	0.684 **	0.730 **	0.611 **	0.887 **	0.743 **
Zn-As	0.274 **	0.044	−0.063	0.076	0.190	0.024	0.110	0.282 *	−0.131	0.158	−0.191 **
Zn-Pb	0.932 **	0.680 **	0.830 **	0.901 **	0.940 **	0.885 **	0.804 **	0.913 **	0.727 **	0.878 **	0.687 **
Zn-Cd	0.805 **	0.789 **	0.752 **	0.855 **	0.780 **	0.706 **	0.789 **	0.786 **	0.810 **	0.885 **	0.793 **
As-Pb	0.222 *	0.194	0.007	0.184 **	0.225 *	0.041	0.203	0.285 *	0.063	0.358 **	−0.173 *
As-Cd	0.023	0.025	−0.072	−0.03 **	0.051	−0.281 *	0.094	0.233 *	−0.24 **	0.190	−0.23 **
Pb-Cd	0.747 **	0.566 **	0.783 **	0.856 **	0.728 **	0.777 **	0.824 **	0.786 **	0.675 **	0.815 **	0.598 **

Note: *, *p <* 0.05; **, *p <* 0.01. DL: Dongli, XQ: Xiqing, JN: Jinnan, BC: Beichen, WQ: Wuqing, BD: Baodi, JH: Jinghai, NH: Ninghe, JZ: Jizhou, UD: Urban district, BH: Binhai New Area.

## Data Availability

Not applicable.

## Supplementary Materials

The following supporting information can be downloaded at: https://www.mdpi.com/article/10.3390/ijerph191610013/s1, Table S1: Land use types at sampling sites; Table S2: Background values and risk screening values of seven heavy metals in soils (mg/kg); Table S3: Classification of geo-accumulation index; Table S4: Classification of the evaluation using Nemerow comprehensive index; Table S5: Classification of pollution load index; Table S6: Principal component analysis and total variance explained content for heavy metal contents in soils and Table S7: Component, rotated component and loading matrix of heavy metal contents in soils of Tianjin based on principal component analysis.
