# Supplementary material for: Accumulation Characteristics and Pollution Evaluation of Soil Heavy Metals in Different Land Use Types: Study on the Whole Region of Tianjin
Source: Int J Environ Res Public Health. 2022 Aug 14;19(16):10013. doi: 10.3390/ijerph191610013 (PMC9408179; doi:10.3390/ijerph191610013)
Supplement: Supplementary file 1 [file ijerph-19-10013-s001.zip › ijerph-1769996-supplementary.pdf]

Table S1 Land use types at sampling sites

Code	Land use types	Detailed information	Number of sampling sites
01	Farmland	Crop land, paddy fields, irrigated land and dry land.	294
02	Orchards	Planting managed perennial woody and herbaceous crops focusing on collecting fruits, leaves, roots, stems, juices, etc.; land for raising seedlings; orchards, tea gardens, etc.	62
03	Woodland	Land of trees, bamboos, and shrubs does not include artificial green forests.	94
04	Grassland	Land where mainly herbaceous plants grow.	10
05	Commercial service land	Land used for commercial and service industries.	7
06	Industrial and mining storage land	mainly used for industrial production and material storage.	51
07	Residential land	urban residential land and rural homesteads.	59
08	Land for public management and public services	Land used for science, education, culture, sanitation, public facilities, etc., including green belts. Among them, primary and secondary schools, hospitals, and parks are applicable to the first-class standards for construction land.	209
09	Special land	such as scenic spots and so on.	5
10	Land for transportation	includes airports, passenger stations, wharfs, highways, etc.	48
11	Water areas and land for water conservancy facilities	include beaches, ditches, swamps, dams, etc.	116
12	Other land	mainly idle wasteland and unused land.	76

Table S2 Background values and risk screening values of seven heavy metals in soils (mg/kg)

Standard	Cr	Ni	Cu	Zn	As	Pb	Cd
Background value in Tianjin	84.2	33.3	28.8	79.3	9.6	21	0.09
Farmland screening value	250	190	100	300	25	170	0.6
Agricultural land control value	1300	—	—	—	100	1000	4.0
Construction land (first category) screening value	3（Cr^{6+}）	150	2000	—	20	400	20

Standard	Cr	Ni	Cu	Zn	As	Pb	Cd
Construction land (first category) control value	30（Cr^{6+}）	600	8000	—	120	800	47
Construction land (second category) screening value	5.7（Cr^{6+}）	900	18000	—	60	800	65
Construction land (second category) control value	78（Cr^{6+}）	2000	36000	—	140	2500	172

Table S3 Classification of geo-accumulation index

I_{geo}	Class	Pollution level
$I_{geo} < 0$	0	No pollution
$0 \le I_{geo} < 1$	1	No pollution to moderate pollution
$1 \le I_{geo} < 2$	2	Moderate pollution
$2 \le I_{geo} < 3$	3	Moderate to severe pollution
$3 \le I_{geo} < 4$	4	Severe pollution
$4 \le I_{geo} < 5$	5	Severe pollution to extreme pollution
$I_{geo} \ge 5$	6	Extreme pollution

Table S4 Classification of the evaluation using Nemerow comprehensive index

P	Pollution level
$P \le 0.7$	Safety
$0.7 < P \le 1.0$	Warning limit
$1.0 < P \le 2.0$	Light pollution
$2.0 < P \le 3.0$	Moderate pollution
$P > 3.0$	Heavy pollution

Table S5 Classification of pollution load index

PLI	Pollution level
PLI ≤1	No pollution
1<PLI ≤2	Light pollution
2<PLI ≤3	Moderate pollution
PLI >3	Heavy pollution

Table S6 Principal component analysis and total variance explained content for heavy metal contents in soils

Component	Initial eigenvalue			Rotated sum of squares loading		
	Total	Variance	Sum (%)	total	Variance	Sum (%)
1	2.646	37.799	37.799	2.009	28.696	28.696
2	1.542	22.030	59.829	1.720	24.578	53.274
3	0.975	13.929	73.758	1.222	17.451	70.725

Component	Initial eigenvalue			Rotated sum of squares loading		
	Total	Variance	Sum (%)	total	Variance	Sum (%)
4	0.801	11.443	85.201	1.013	14.476	85.201
5	0.463	6.613	91.814			
6	0.297	4.249	96.063			
7	0.276	3.937	100.00			

Table S7 Component, rotated component and loading matrix of heavy metal contents in soils of Tianjin based on principal component analysis

Heavy	Component				Rotated component				Component score			
mental	PC1	PC2	PC3	PC4	PC1	PC2	PC3	PC4	PC1	PC2	PC3	PC4
Cr	0.600	0.679	-0.055	-0.166	0.085	0.893	0.149	0.158	-0.047	0.536	-0.040	0.027
Ni	0.586	0.638	-0.148	-0.307	0.097	0.922	0.085	-0.001	-0.028	0.593	-0.126	-0.146
Cu	0.577	0.043	-0.478	0.582	0.088	0.185	0.927	-0.032	-0.202	-0.064	0.879	0.004
Zn	0.729	-0.316	-0.060	0.261	0.617	0.050	0.566	0.010	0.212	-0.125	0.405	0.056
As	0.094	0.460	0.763	0.437	-0.016	0.115	-0.027	0.989	0.011	-0.080	0.024	0.996
Pb	0.713	-0.437	0.312	-0.145	0.898	0.048	0.079	0.052	0.505	-0.054	-0.159	0.070
Cd	0.748	-0.412	0.196	-0.247	0.893	0.140	0.075	-0.078	0.493	0.030	-0.189	-0.079
